# Twelve-Week Daily Consumption of *ad hoc* Fortified Milk with ω-3, D, and Group B Vitamins Has a Positive Impact on Inflammaging Parameters: A Randomized Cross-Over Trial

**DOI:** 10.3390/nu12113580

**Published:** 2020-11-22

**Authors:** Morena Martucci, Maria Conte, Laura Bucci, Enrico Giampieri, Cristina Fabbri, Maria Giustina Palmas, Massimo Izzi, Stefano Salvioli, Angelo Vittorio Zambrini, Carla Orsi, Patrizia Brigidi, Aurelia Santoro, Miriam Capri, Daniela Monti, Claudio Franceschi

**Affiliations:** 1 Department of Experimental, Diagnostic and Specialty Medicine, University of Bologna, 40138 Bologna, Italy; m.conte@unibo.it (M.C.); laura.bucci2@unibo.it (L.B.); enrico.giampieri@unibo.it (E.G.); cristina.fabbri12@unibo.it (C.F.); stefano.salvioli@unibo.it (S.S.); aurelia.santoro@unibo.it (A.S.); miriam.capri@unibo.it (M.C.); claudio.franceschi@unibo.it (C.F.); 2 Alma Mater Research Institute on Global Challenges and Climate Change (Alma Climate), University of Bologna, 40126 Bologna, Italy; mariagiustina.palmas@unibo.it (M.G.P.); massimo.izzi@unibo.it (M.I.); 3 Department of Quality, Innovation, Safety, Environment, Granarolo S.p.A., 40057 Bologna, Italy; vittorio.zambrini@granarolo.it (A.V.Z.); carla.orsi@granarolo.it (C.O.); 4 Unit of Microbial Ecology of Health, Department of Pharmacy and Biotechnology, University of Bologna, 40126 Bologna, Italy; patrizia.brigidi@unibo.it; 5 Department of Experimental and Clinical Biomedical Sciences “Mario Serio”, University of Florence, 50134 Florence, Italy; daniela.monti@unifi.it; 6 Department of Applied Mathematics, Institute of Information Technology, Mathematics, and Mechanics (ITMM), Lobachevsky State University of Nizhny Novgorod—National Research University (UNN), 603950 Nizhny Novgorod, Russia

**Keywords:** aging, fortified milk, homocysteine, ω-6/ω-3 ratio, ω-3 index

## Abstract

Background and Aim: A state of chronic, subclinical inflammation known as inflammaging is present in elderly people and represents a risk factor for all age-related diseases. Dietary supplementation with *ad hoc* fortified foods seems an appealing strategy to counteract inflammaging. The purpose of this study was to test the efficacy of elderly-tailored fortified milk on inflammaging and different health parameters. Methods: A double-blind randomized cross-over study was performed on forty-eight volunteers aged 63–80 years. The fortified milk was enriched with ω-3 polyunsaturated fatty acids (eicosapentaenoic acid, EPA; docosahexaenoic acid, DHA), vitamins (25-hydroxyvitamin D, E, C, B6, B9, B12), and trace elements (zinc, selenium). The two intervention periods lasted for 12 weeks, with a 16-week washout intermission. Results: Compared to placebo, the consumption of fortified milk increased the circulating levels of different micronutrients, including vitamins and the ω-3 index of erythrocyte membranes. Conversely, it reduced the amount of arachidonic acid, homocysteine, and ω-6/ω-3 ratio. Conclusion: Twelve-week daily consumption of *ad* *hoc* fortified milk has an overall positive impact on different health parameters related to inflammaging in the elderly.

## 1. Introduction

Italy is the second country in the world for the number of elderly people (>65 years), with an estimated 172.9 people over 65 per 100 young people, according to data reported on 1 January 2019 by the National Institute of Statistics (ISTAT) [1]. People aged over 65 years old show one or more chronic pathologies, which are projected to increase in number due to the elongation of life expectancy. This rising trend anticipates an alarming and unsustainable chronic disease burden as well as associated healthcare costs in developed countries [2]. Therefore, strategies to preserve as much as possible the health span, preventing/postponing the onset of age-related diseases and geriatric syndromes should be prioritized, as suggested by the World Health Organization [3]. Among possible preventative strategies, a healthier diet represents the pillar of lifestyle interventions, as nutrition is one of the major modulators of molecular mechanisms that underpin “inflammaging” [4,5]. Inflammaging is a chronic low-grade inflammatory status associated with age [6,7], representing *per se* a robust predictor of morbidity and mortality when inflammatory hallmarks largely overcome the anti-inflammatory counterpart [8,9]. Therefore, counteracting inflammaging could prevent/decelerate all age-associated diseases [10].

In the present study, we aimed to test the efficacy of a nutritional intervention intended to counteract inflammaging. Such intervention was composed by *ad hoc* fortified milk containing a cocktail of micronutrients with anti-inflammatory and antioxidant activities, which are usually deficient in old age [11,12,13]. The choice of milk has a double advantage: (i) its molecular characteristics have been proven to allow a proper micronutrient absorption [14,15,16,17]; (ii) being a drink, it avoids chewing problems in old people, also facilitating digestive functions. The selected micronutrients are the following: ω-3 polyunsaturated fatty acids (PUFA), eicosapentaenoic acid (EPA) and docosahexaenoic acid (DHA), the 25-hydroxyvitamin D [25(OH)D], C, E, and B group vitamins, as well as zinc and selenium trace elements. This choice meets with nutritional guidelines, which encourage the dietary intake of “good fats”, in particular, DHA and EPA, recognized for their powerful anti-inflammatory properties, especially on the brain and heart [18,19,20]. Vitamin 25(OH)D deficiency is the most prevalent hypovitaminosis worldwide among old individuals, including Italy [21]. In particular, two Italian regions, i.e., Emilia Romagna (where the recruitment was performed) and Veneto, register a vitamin D deficiency of 36% among the age range 61–75, which is probably due to reduced time spent outdoors [22]. In recent years, this hypovitaminosis has been associated with a plethora of pathophysiological conditions, overcoming its established role in muscle/bone homeostasis and becoming an even more crucial micronutrient to counteract inflammaging and age-related diseases [23,24]. Similarly, C and E vitamins, together with zinc and selenium, show a tendency to decrease in old adults. Their replenishing/maintenance is fundamental for strengthening the immune system through their strong anti-oxidative properties [25,26,27,28]. Lastly, group B vitamins are strongly involved in the prevention of age-related dysfunction, lowering the hyperhomocysteinemia, which is an independent risk factor for the onset of neurological and cardiovascular diseases [29].

In the present study, an interventional trial with a double-blind cross-over experimental design was performed on 48 elderly volunteers. We tested the efficacy of a relatively short nutritional intervention based on milk fortified with the above-mentioned micronutrients at concentrations complying with the international guidelines [30]. A variety of clinical trials based on enriched milk or dairy products have been performed until today, but most of them were focused on a specific pathological condition/nutritional deficiency in different age classes. At variance, the present trial was focused on elderly subjects and their nutritional needs adopting the approach of the cocktail, instead of the single micronutrient, aimed at favoring potential synergistic effects [31]. The main goal was to develop a nutritional strategy aimed at preventing or reducing common micronutrient deficiencies and counteracting inflammaging, one of the major risk factors for virtually all age-associated diseases in old adults. In particular, the primary objectives were to verify if the fortified milk supplied the micronutrients, evaluating their circulating levels and if it offered benefits on inflammaging by the modulation of some prototypical inflammatory parameters. A secondary objective was to evaluate if the fortified milk was able to improve the general health status by a comprehensive approach that considered hemato-biochemical markers, functional status, cognitive functions, physical performance, and mood state.

## 2. Materials and Methods

### 2.1. Ethics Approval

All procedures of the study protocol were approved by the local Ethical Committee of S. Orsola-Malpighi Hospital in Bologna (n° 7/2013/U/Sper issued on 15 January, 2013) and written informed consent was obtained from each participant. The trial was conducted in accordance with the Helsinki Declaration, and it was registered on the National Institute of Health Clinical Trials (clinicaltrials.gov. Identifier: NCT02027285).

### 2.2. Study Population

Community-dwelling elderly volunteers, representative of the general population, were recruited at our research laboratory by trained research personnel between February 2013 (baseline characterization) and December 2013 (end of the treatment). The identification of potential volunteers was performed through advertisements and promotional leaflets posted in medical offices, pharmacies or socio-cultural centers from our team. Accordingly, interested volunteers contacted the study team for further information and a basic assessment of eligibility. All volunteers received detailed written information, and many of them were invited to discuss the study, either face-to-face or over the telephone, with a member of the study team. Following the exchange of information and basic eligibility questions, the volunteer underwent the next stage of recruitment if he/she was still interested in participating, and if deemed eligible. Overall, 48 old adults (26 males and 22 females, mean age 70 ± 4.5 years) out of 53 subjects screened for eligibility met inclusion criteria (5 subjects were excluded). Inclusion criteria were the following: (i) age between 63 and 80 years; (ii) free-living status, resident in Bologna province (Emilia Romagna region, Italy); (iii) capability to express informed consent. Exclusion criteria were the following: (i) allergy or intolerance to cow’s milk; (ii) presence of other concurrent dietary interventions and/or using of supplements with the same micronutrients proposed in the trial; (iii) presence of celiac disease or intestinal malabsorption syndromes, cancer, type I diabetes, chronic viral hepatitis, chronic-degenerative diseases, chronic therapy with anticoagulants (except cardioaspirin), corticosteroids. The characteristics of the investigated cohort are reported in Table 1.

### 2.3. Study Design

**Figure 1:**
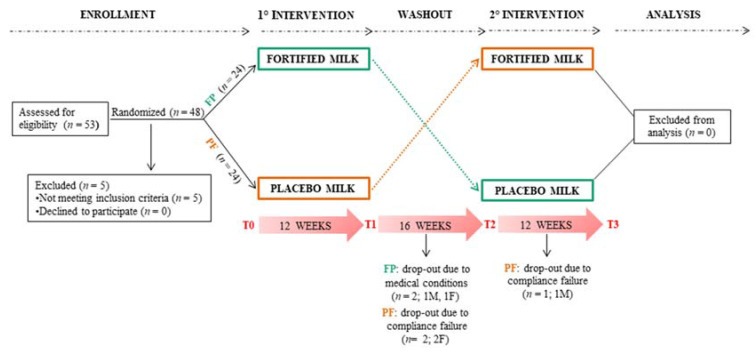
Flow chart of the 40-week study protocol with cross-over. Fifty-three subjects were assessed for eligibility for the study and 5 out of 53, not meeting inclusion criteria, were excluded. Forty-eight subjects were randomly allocated to the two study arms. One arm started the study with consumption of the fortified milk, while the other one started the study with the placebo milk and then each group was assigned to the opposite treatment. Both the treatment periods were 12 weeks long with 16 weeks of washout in between. Overall, five subjects left the study for medical conditions or failure to comply, four subjects left the study during the washout period, and one left the study after the washout. Volunteers were assessed at the baseline (T0) and the end of the first intervention (T1), the washout (T2), and the second intervention (T3). FP (green line) = arm: participants on fortified milk and then placebo, PF (orange line) = arm: participants on placebo and then fortified milk.

The interventional trial was set up on a double-blind cross-over experimental design. Neither the participants nor the researchers knew the administered treatment, thus avoiding biases, and all subjects crossed-over throughout all the treatment groups after a washout period in between. Forty-eight participants were enrolled and randomly allocated to the two study arms to a 1:1 ratio after stratification by gender and age. Randomization was performed by entering the gender and age of a subject into a software expressly created and implemented by our team for this study and others, which automatically randomly allocates and generates a unique ID code. The two arms were nominated as FP (volunteers who initially drank the fortified milk and then the placebo) and PF (volunteers who initially drank the placebo milk and then the fortified one), respectively. Treatment periods of 12 weeks were separated by 16 weeks of washout. After the washout period, each group was assigned to the opposite treatment. During the admission time, the medical history and medications of each volunteer were collected to evaluate their eligibility for the study. Subsequently, subjects enrolled were assessed at the times: (i) baseline or T0; (ii) end of the first treatment or T1; (iii) end of the washout period or T2; (iv) end of the second treatment or T3. At each of these times, blood samples as well as questionnaire data on cognitive and physical status were collected. New medications or disorders that occurred during the study were punctually recorded. Figure 1 shows the experimental design and the flow chart of the study protocol.

### 2.4. Intervention

The intervention consisted in the drinking of 250 mL of cow’s milk daily, to be consumed at room temperature during the breakfast meal. The two kinds of milk appeared identical, with encrypted codes for participants and researchers. In particular, they shared the Ultra-High-Temperature (UHT) and the high digestibility (lactose < 0.5%). The principal difference was the micronutrient supplementation complying with the Population Reference Intake (PRI) specific for the age range of the study population, as recommended by the national guidelines “Livelli di Assunzione di Riferimento di Nutrienti ed energia per la popolazione italiana (LARN)” [29]. Micronutrient supplementation increased the fortified milk caloric intake of 27 Kilocalories per day with respect to the placebo milk. Vitamin C was added to the milk as an antioxidant to prevent oxidative deterioration of fats; thus, it was not considered as a bioavailable micronutrient.

Milk was delivered to the volunteers at the research laboratory site. During the intervention periods, compliance with the consumption protocol was monitored by weekly telephone calls and collection of the emptied containers. The complete composition of the formulated dairy products, produced by an Italian dairy Company (Granarolo S.p.A., Bologna, Italy), is reported in Table 2.

### 2.5. Data Collection

Data collection was assessed at each time point (T0, T1, T2, and T3) of the study design. A standardized questionnaire, including socio-demographic informations, lifestyle, health and morbidities (present and past diseases, prescribed medicines), and anthropometric measurements (i.e., height, weight, waist and hip circumference, body mass index (BMI)) was administered to the participants by a trained nurse/researcher through a face-to-face interview. All tests included in the questionnaire were pen-and-paper testing performed at the laboratory of our team in Bologna. In particular, functional status was assessed by Activities of Daily Living scale (ADL) (scores ranging from 0, all functions lost, to 6, all functions preserved) [32] and Instrumental Activities of Daily Living scale (IADL) (scores ranging from 0, all functions lost, to 8, all functions preserved) [33]. Physical performance was assessed by Short Physical Performance Battery (SPPB), which explores gait speed, muscle strength and balance with a performance score ranging 0–12 [34]. A Handgrip Strength Test was performed to measure the maximum isometric strength of the dominant hand, using a hand-held dynamometer (Smedley’s dynamometer, Scandidact, Kvistgaard, Denmark). Cognitive status was assessed by the Stroop Color-Word Interference Test (STROOP), whose scores were adjusted by gender and education [35] as well as the Standardized Mini-Mental State Examination test (SMMSE) [36], whose scores were adjusted by age and education [37].

The STROOP test is a mainstay of research on age-related differences in selective attention, automaticity, inhibitory processes, and executive control. Subjects were instructed to read three different subtasks (cards) of 40 stimuli each as quickly and accurately as possible. Two of them represent the “congruous condition” i.e., to read the names of the colors both printed in black ink (card 1) and through color patches (card 2). Conversely, card 3 involves “incongruent condition”, color-words are printed in an inconsistent color ink (for instance the word “red” is printed in green ink). There was no time limit to complete each subtask, and the outcome variables were the time of reaction recording in seconds and number of error responses in performing each card [35]. A corrected score for the number of errors related to cards 2 and 3 was calculated, respectively. For each error of the card, twice the average reading time per word was added to the reading time of the card and multiplied for the total number of errors made in that card. Furthermore, the interference effect of words upon the naming of colors was assessed by calculating the difference between card 3’s corrected score and card 2’s corrected score [38].

The SMMSE is the most widely used cognitive tool for its short duration and high reproducibility. It consists of 11 task items that assess the cognitive domains of visuospatial skills, language, concentration, working memory, memory recall, and orientation with a maximum score of 30. A score < 24 is the generally accepted cutoff indicating the presence of cognitive impairment [36]. Depression was investigated by Geriatric Depression Scale short form (GDS, 15 items) [39] and self-perceived health was evaluated by the EuroQol-5D test (EQ-5D) [40].

### 2.6. Blood Sampling and Hematological–Biochemical Measurements

Blood collection was performed at each time point, in a range of 5 days within the time point (T0, T1, T2, and T3). Fasting blood samples were drawn by venipuncture in the morning and processed 3 h from being withdrawn. Serum was obtained after clotting and centrifugation at 760× *g* for 20 min at 4 °C; plasma was separated by centrifugation at 2000× *g* for 20 min at 4 °C. Both plasma and serum were rapidly frozen and stored at −80 °C until the analysis.

Glycaemia, creatinine, alanine aminotransferase (ALT), aspartate aminotransferase (ASP), C-reactive protein (CRP), homocysteine, total and HDL cholesterol and triglycerides were measured in fresh serum; the concentration of LDL cholesterol was calculated by using the Friedewald equation: LDL-C (mg/dL) = total cholesterol − HDL-C − (triglycerides/5). Folate, vitamin B12, zinc, and selenium were measured in frozen serum, while vitamin B6 and vitamin E were measured in frozen plasma. All these analyses were performed by the clinical laboratory at the accredited Nigrisoli Hospital (Bologna, Italy) with high-quality standards.

Serum insulin and total 25(OH)D were measured by a chemiluminescent immunoassay (LIAISON^®^ DiaSorin, Saluggia, Italy) and analyzed by the LIAISON^®^ Analyzer. Insulin resistance status was assessed by the homeostasis model assessment of insulin resistance (HOMA-IR) according to the following formula: insulin (μU/mL) × glucose (mg/dL)/405.

### 2.7. Lipidomic Assay

Erythrocyte membrane fatty acid profile analysis was carried out by gas chromatography starting from the erythrocyte membrane pellets and performed in a custom service fashion by a facility of the National Research Council (Bologna, Italy) as previously described [41]. The following fatty acids were measured: saturated fatty acids (SFA) (palmitic acid 16:0, stearic acid 18:0), monounsaturated fatty acids (MUFA) (palmitoleic acid 16:1, oleic acid 9c 18:1, vaccenic acid 11c 18:1), polyunsaturated fatty acids (PUFA) (ω-6: linoleic acid 18:2, dihomo-γ-linolenic acid (DGLA) 20:3, arachidonic acid 20:4; ω-3: eicosapentaenoic acid (EPA) 20:5, docosahexaenoic acid (DHA) 22:6), and trans-unsaturated fatty acids (TFA) (Trans 18:1, Trans 20:4).

### 2.8. Cytokine Measurement

Plasma levels of IL-6 and IL-10 were measured by high sensitivity enzyme-linked immunosorbent assay (ELISA) (America-IBL), according to the manufacturer’s instructions. The choice of these two cytokines reflects their opposite role in inflammaging setting. In particular, elevated IL-6 pro-inflammatory levels are associated with a reduced capacity to reach the extreme limits of human life, whereas high levels of anti-inflammatory IL-10 are present among centenarians [42].

### 2.9. Statistical Analyses

Power analysis (80% power, two-sided alpha = 0.05, beta > 0.95; software: G*POWER 3.1) for detecting at least the significant change of a variable after the intervention, resulted in an adequate sample size of 44 participants.

To assess differences between the two arms (FP, PF), we performed time-series analysis (T0, T1, T2, T3), considering both arms separately. Since the two arms showed similar levels of the micronutrients at baseline and after the washout period, data analysis was mainly performed on the two assembled groups. All data are reported as mean ± SD (Standard Deviation) in figures and tables, and the significance was tested by Fisher’s exact test for contingency matrices, one-way ANOVA test, and linear mixed effect model analysis for non-independent/multilevel data. All *p*-values were collectively corrected for multiple testing using a false discovery rate correction method (Benjamini–Hochberg), obtaining q-values (or corrected p-values based on the threshold of 0.05) reported in the data analysis. Box plots in figures are reported with gray lines for the best comparison of data (FP vs. PF) at the single volunteer level.

The linear mixed effect model included three fixed terms and a random effect on the intercept: a numerical term for the time point of the study (from 0 to 3, to account for seasonality drift), a binary term for the effect of the milk consumption (at time 1 and 3 for both arms), and a binary term for the consumption of the fortified formula (at time 1 for the FP arm and time 3 for the PF arm). No interaction between the terms was included. The model would be represented as “value ~ 1|subject + time + milk + fortified”. Primary and secondary outcomes of the study were analyzed using the same statistical approach above described. In particular, primary outcomes have been the assessing of circulating micronutrients and cytokines’ levels after the consumption of the fortified milk. Meanwhile, secondary outcome has been a comprehensive evaluation of the health status by the measurement of biological markers (hematological-biochemical variables, homocysteine, ω-6/ω-3 ratio, ω-3 index), functional status (ADL, IADL), physical performance (SPPB, Handgrip Strength Test) cognitive functions (STROOP, SMMSE) and mood state (GDS, EuroQol-5D test).

## 3. Results

### 3.1. Baseline Characteristics of the Study Population

Milk was well tolerated and appreciated by participants, leading to a high compliance (89.6%). All volunteers completed the first intervention period (T1), while four subjects left the study during the washout period (T2 not completed), and one subject left the study after the washout (T3 not completed). Motivations included the onset of new medical conditions matching exclusion criteria or compliance failure, as reported in Figure 1. Baseline characteristics of anthropometric measurements and the main metabolic and health parameters of the two arms (FP, PF) are shown in Table 3.

The FP and PF arms showed similar levels of the micronutrients at baseline and after the washout period, as illustrated in Table 4.

### 3.2. Circulating Micronutrient Levels

At baseline, participants did not show vitamin deficiencies, except for vitamin 25(OH)D (18 ± 10 ng/mL), as shown in Table 5. A significant increase of several vitamins after the treatment: i.e., B9 (q < 0.0001), B6 (q **=** 0.015), B12 (q **=** 0.0001), including the 25(OH)D vitamin (q = 0.0002) was observed after intervention with the fortified milk. Interestingly, vitamin 25(OH)D increased its concentration up to 23 ng/mL (29%) in 12 weeks of treatment. Conversely, vitamin E showed only a trend to increase (q = 0.110).

No significant change occurred after the treatment for the circulating levels of zinc and selenium minerals (q = 0.786 and q = 0.106, respectively) (Table 5).

### 3.3. Lipidomics

To evaluate the lipid profile, a lipidomic analysis was performed on the total lipid composition (TFA, SFA, MUFA, PUFA) of the erythrocyte membranes, including linoleic acid, DGLA acid, arachidonic acid, DHA, and EPA (Table 6). The erythrocytes contain all the classes of fatty acids and given their activity of transport in all organism districts, they are considered “reporters” of the general content of fatty acids [41,43].

**Figure 2:**
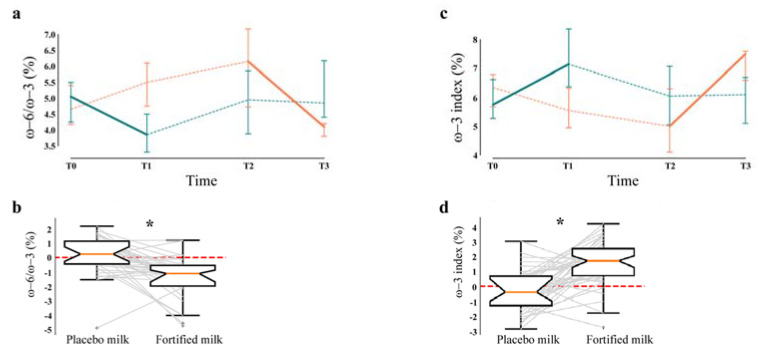
Ratio of ω-6/ω-3 PUFA and ω-3 index. (**a**) Time-series visualization of ω-6/ω-3 PUFA ratio in participants of both arms. (**b**) Boxplot of the variation of ω-6/ω-3 ratio in participants on placebo vs. those on fortified milk is displayed (q < 0.0001 *). (**c**) Time-series visualization of ω-3 index (EPA + DHA content in erythrocytes on total fatty acids) in participants of both arms. (**d**) Boxplot of the variation of ω-3 index in participants on placebo vs. those on fortified milk is displayed (q < 0.0001 *). Statistical analysis was performed by linear mixed effect model and a q-value (corrected p-value for the Benjamini–Hochberg multiple comparisons) < 0.05 was considered statistically significant. FP (green line) = arm: fortified milk and then placebo, PF (orange line) = arm: placebo and then fortified milk, Solid line = fortified milk, Dashed line = placebo milk/washout, DHA = docosahexaenoic acid, EPA = eicosapentaenoic acid.

At baseline, participants showed lipid levels of erythrocyte membranes (Table 6) within the normal range. Concerning the ω-3 PUFA, the results showed a significant increase of DHA (q < 0.0001) and EPA levels (q = 0.040) after the intervention period. A significant decrease of arachidonic acid (q = 0.001), belonging to the PUFA ω-6 series, was also observed. The ω-6 to ω-3 ratio, calculated as percentage of the total fatty acid content of erythrocyte membranes, was also evaluated, and a significant reduction from 5% to 4% emerged (Figure 2, panels a/b, q < 0.0001). Accordingly, the “ω-3 index”, representing the percentage of EPA and DHA amount (ω-3 PUFA) on the total fatty acid content of erythrocyte membranes, significantly increased after the fortified milk consumption from 5.6 to 7.3% (Figure 2, panels c/d, q < 0.0001).

### 3.4. Hematological–Biochemical Assessment

Blood biochemical parameters, i.e., HDL cholesterol, LDL cholesterol, triglycerides, glycemia, HOMA-IR, creatinine, fibrinogen, ALT, ASP, and homocysteine, were within the normal reference ranges at baseline, and the majority of these parameters did not show significant modifications after the intervention. Conversely, total cholesterol was slightly over the normal threshold at baseline and after fortified milk consumption, but not significantly. The hepatic enzymes ALT and ASP slightly increased their levels after the treatment (q = 0.019, q = 0.0006 respectively), but remaining within the normal range (8–40 U/L) (Supplementary Materials Table S1).

**Figure 3:**
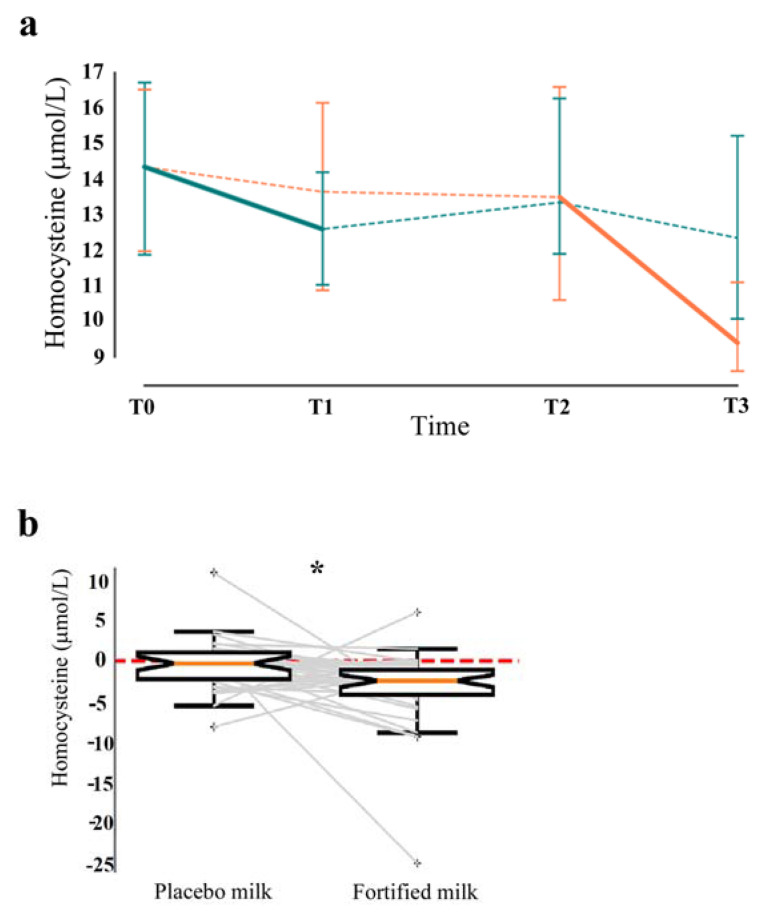
Circulating homocysteine levels. (**a**) Time-series visualization of circulating homocysteine levels in participants of both arms. (**b**) Boxplot of the variation of homocysteine in participants on placebo vs. those on fortified milk is displayed (q < 0.0001 *). Statistical analysis was performed by linear mixed effect model and a q-value (corrected p-value for the Benjamini–Hochberg multiple comparisons) < 0.05 was considered statistically significant. FP (green line) = arm: participants on fortified milk and then placebo, PF (orange line) = arm: participants on placebo and then fortified milk, Solid line = fortified milk, Dashed line = placebo milk/washout.

On the contrary, circulating homocysteine concentration significantly decreased after fortified milk consumption from 15.2 to 11.6 µmol/L (Figure 3, panels a/b, q < 0.0001).

### 3.5. Inflammatory Profile

Cytokines and CRP displayed low circulating concentrations both before and after the intervention without significant changes (Supplementary Materials Table S2).

### 3.6. Physical, Cognitive, and Mood Evaluation

Functional and physical status was evaluated by the administration of different tests, i.e., ADL, IADL, SPPB scales, and handgrip test. No changes after the treatment were observed (data not shown). Moreover, we evaluated cognitive interference, mood disorders, and self-perceived health using STROOP, GDS, and EQ-5D-EuroQol-5D scales, respectively. No significative difference after the treatment emerged (data not shown). Interestingly, the assessment of cognitive status using SMMSE displayed a trend to increase after fortified milk consumption, although this assessment was not significant (Supplementary Materials Figure S1).

## 4. Discussion

The present study aimed to reduce/postpone inflammaging by a nutraceutical approach according to the Geroscience objectives [10,44], whose main goals are to develop feasible, practical, and safe interventions to fight multiple chronic diseases and slowing the aging processes in the general population (real world) [9]. In this context, the main results obtained were the significant increase of different micronutrients added to the fortified milk as group B vitamins, vitamin D, DHA, EPA, and a significant positive modulation of other biological parameters i.e., an increasing in ω-3 index and a reduction in ω-6/ω-3 ratio as well as in the amount of circulating homocysteine.

In particular, circulating levels of B9, B6, and B12 vitamins increased concomitantly with the reduction in homocysteine. Accordingly, B vitamins are involved in homocysteine metabolism, and their concentrations are inversely related to homocysteine [45,46]. The maintenance of low levels of circulating homocysteine is an important goal in elderly adults. Hyper-homocysteinemia is recognized *per se* as a major risk factor for a variety of cerebrovascular and neurodegenerative diseases, such as coronary heart disease, stroke, cognitive impairment, dementia, and Alzheimer’s disease [45,47,48,49,50,51,52,53]. Data regarding the impact of circulating homocysteine on brain volume and functions suggest that high levels of homocysteine are associated with both brain volume loss and accelerated brain atrophy in old people without cognitive impairments [54,55,56]. An increase in the circulating homocysteine greater than 14 µmol/L can double the risk of Alzheimer’s disease [49]. Moreover, an association between high homocysteine levels and a worsening in sensory and motor peripheral nerve function has been reported in a cohort of 678 subjects followed for 6 years in the InCHIANTI longitudinal study [57]. Accordingly, supplementation with B vitamins has been suggested as an effective therapeutic approach to preserve brain functions by lowering circulating homocysteine [58,59,60,61].

Vitamin 25(OH)D deficiency is a major medical issue, particularly in the elderly, as we reported previously in various research studies, including centenarian studies [62,63]. The significant increase in vitamin 25(OH)D levels observed after fortified milk consumption demonstrates that the fortified milk is capable of effectively supplying this important vitamin, whose deficiency is the most common in elderly people, due to insufficient intake from diet and scarce sun exposure [64,65]. The vitamin 25(OH)D is predominantly known for its pivotal functions in calcium homeostasis and bone metabolism, preventing the risk of muscle weakness and fractures [66]. Our previous European project on the elderly population called Nu-Age (“New dietary strategies addressing the specific needs of the elderly population for healthy aging in Europe”), a one-year elderly-tailored intervention based on a Mediterranean diet supplemented with vitamin 25(OH)D, demonstrated a reduction of the bone loss rate in elderly Europeans [67,68]. Beyond its key role in musculoskeletal health, vitamin 25(OH)D deficiency influences many other pathophysiological pathways/diseases including the immune system, cognitive functions, diabetes, cardiovascular diseases, and cancer [23,24]. Therefore, vitamin 25(OH)D supplementation becomes crucial in elderly individuals, and it should be continuously administrated [69]. The Task Force for the Clinical Guidelines Subcommittee of the Endocrine Society suggests 30–60 ng/mL as an optimal range to sustain bone health in elders [70,71], whereas a cutoff <10 ng/mL indicates a severe deficiency [21]. The present intervention has successfully increased the circulating vitamin 25(OH)D levels, and a prolonged treatment could likely contribute to reach and maintain the desired serum levels.

At variance, vitamin E is the only one that did not increase after the treatment, which is likely since its baseline level was close to the upper reference limit of the normal range. A further explanation could be an impairment of the intestinal absorption process. The vitamin E absorption efficacy can vary from 10% to 95% due to genetic alterations influencing both the metabolism and the factors regulating its bioavailability [72,73,74]. Moreover, evidence showed that vitamin E absorption can be influenced by the competition with other micronutrients such as vitamin 25(OH)D [75].

As far as trace elements are concerned, the study population showed proper levels of zinc and selenium at baseline, and no changes after the intervention were observed. The current knowledge of selenium metabolism still has to be elucidated [76] conversely to zinc metabolism. In general, micronutrient absorption may be reduced/blocked by interaction with other nutrients acting as inhibitors/competitors [77,78]. Overall, nutritional competitors and/or normal amounts of zinc and selenium may have interfered with the assimilation of these trace elements.

Due to their anti-inflammatory properties, ω-3 PUFA have been suggested as therapeutic agents to target inflammaging [79]. We first documented a remodeling of erythrocyte cell membranes after the treatment. Then, the lipidomic analysis underlined that the daily consumption of the fortified milk allowed a significant augment in ω-3 PUFA content compared to the ω-6 PUFA counterpart after 12 weeks of treatment. In particular, arachidonic acid decreased, while ω-3 DHA and EPA increased (q < 0.0001, q = 0.040 respectively). This change reduced the ω-6/ω-3 ratio to 4% (q < 0.0001), considering that the optimal range is between 3.5 and 5.5%. Moreover, the ω-3 index, considered an independent cardiovascular risk marker in primary prevention [80], significantly increased after the intervention from 5.6 up to 7.3% (q < 0.0001), getting very close to the level suggested for a good cardioprotective effect (≥8%), while an index ≤ 4% has been associated with a high cardiovascular risk [81,82]. Importantly, the literature suggests the relevance of the ω-6/ω-3 ratio in correlation with human health status. Specifically, a ratio of 4:1 has been associated with a 70% mortality decrease in the secondary prevention for heart morbidities [83], while a 3:1 ratio (or even 1:1) has been suggested to prevent the risk of many chronic diseases [83,84], including those related to the brain and cardiovascular system [18,85].

Additionally, we checked whether the fortified milk could have effects on physical, cognitive, and functional performances, as well as mood state with not significant results. Overall, only cognitive status detected through SMMSE showed a trend to improve after the intervention, although this was not significant due to a great interindividual variability. It is plausible to speculate that the interaction of the micronutrients present in the fortified milk may have a positive impact on cognitive function in longer treatment times, activating different metabolic pathways in an additive or synergistic fashion. There is a general consensus that these micronutrients can positively impact on learning and memory abilities. A dietary deficiency of ω-3 fatty acids in humans has been associated with increased risk of several mental disorders, including attention-deficit disorder, dyslexia, dementia, depression, bipolar disorder, and schizophrenia [86]. Moreover, a recent literature revision described a reduced intake of B group vitamins and vitamin D among those micronutrient deficits able to affect cognitive decline [13]. Evidence demonstrated that vitamin B12 and B9 deficiencies are strongly associated with mental health decline in old persons, while a severe deficiency of vitamin D (<10 ng/mL) represents a risk factor for cognitive disorder development such as dementia and Alzheimer’s disease [87].

Since all vitamins and trace elements added to the fortified milk are known modulators of the inflammatory status, especially vitamin D [88,89], we evaluated their effect on some prototypical markers of inflammation (C-reactive protein [CRP], IL-6) and anti-inflammation (IL-10). No effect was observed on these parameters; however, it is worth mentioning that the levels of these mediators were already very low at baseline. Still, it is possible that the micronutrients (vitamins including vitamin D and ω-3 PUFA) with anti-inflammatory properties may help in decreasing the inflammatory state of patients with more elevated levels of inflammation. This hypothesis needs to be further tested.

The strengths of the study are the following: (i) the recruited elderly volunteers are representative of the general population (real world); (ii) the proposed fortified milk is suitable for all old adults avoiding chewing and digestive problems with a very low percentage of lactose (<0.5%); (iii) a high compliance was reached due to the palatability of the fortified milk; (iv) the cross-over experimental design allowed doubling the sample size, being the subjects of both groups very similar at baseline and after the washout. Conversely, the main limitation of the study is represented by the slightly higher caloric intake of the fortified milk with respect to the placebo one (27 kilocalories more per day). Weight change evaluation was performed along the trial, registering a weight average increase equal to 0.14 kg associated to the fortified milk consumption, but no significant difference with respect to the placebo milk was observed (p value = 0.73).

To note, the study was conducted from February to December with a high variability in sun exposure and fresh fruit as well as vegetable availability. However, the possible seasonality influence on micronutrient levels was considered by the applied statistical model.

## 5. Conclusions

In conclusion, our results indicate that the fortified milk with the “formula” developed for this study is promising in replenishing the most widespread micronutrient deficiencies in old people and in improving parameters related to cerebrocardiovascular health (homocysteine, ω-3 index). We surmise that this approach may be an attractive nutritional strategy to prevent inflammaging by using a high compliance cocktail of anti-inflammatory molecules usually deficient in the elderly.

## Figures and Tables

**Table 1:** Baseline diseases/medications of the study population.

Diseases	Baseline FP (*n* = 24) PF (*n* = 24) (*n*, %)		q Value
Neuropsychological disorders (anxiety/depression/insomnia)	4 (17%)	5 (21%)	1
Hypertension	15 (63%)	9 (38%)	1
Hypercholesterolemia	2 (8%)	6 (25%)	1
Cardiovascular disorders (rhythm disturbances/thrombosis)	6 (25%)	6 (25%)	1
Musculoskeletal system syndromes (arthrosis/hiatal hernia/osteoporosis/fibromyalgia/joint gout)	3 (13%)	4 (17%)	1
Chronic respiratory diseases (asthma/chronic obstructive pulmonary disease)	0 (0%)	2 (8%)	1
Digestive disorders (ulcerative esophagitis/gastroesophageal reflux/gastritis)	5 (21%)	4 (17%)	1
Type 2 diabetes	1 (4%)	1 (4%)	1
Prostatic hyperplasia/hypertrophy	4 (17%)	3 (13%)	1
Ocular hypertension/glaucoma	2 (8%)	1 (4%)	1
Hypothyroidism/hyperthyroidism	3 (13%)	3 (13%)	1
Medications (*n*) ^a^	3 ± 2	3 ± 2	1

^a^ Expressed as mean ± standard deviation (SD), The comparison between the two arms (FP vs. PF) was performed by using Fisher exact test and one-way ANOVA test with Benjamini–Hochberg correction, considering q (corrected p-value) < 0.05 statistically significant. No significant difference emerged between the two arms. FP = arm: participants on fortified milk and then placebo, PF = arm: participants on placebo and then fortified milk.

**Table 2:** The nutritional label of the two kinds of milk used in the study.

	Placebo Milk			Fortified Milk		
	100 mL	250 mL	%LARN (250 mL)	100 mL	250 mL	%LARN (250 mL)
**Energy (kcal)**	46	115	-	57	142	-
**Protein (g)**	3.10	7.75	12%	4	10	15.5%
**Total Carbohydrates (g)**	4.9	12.25	20%	5.6	14	22.9%
Dietary fiber (g)	0	0	0%	0	0	0%
Sugars (g)	4.9	12.25	20%	5.6	14	22.9%
Lactose	<0.5	<1.25	-	<0.5	<1.25	-
**Total fat (g)**	1.6	4	20%	2.04	5.1	25.5%
Saturated fat (g)	1.2	3	5%	1.27	3.2	5.3%
DHA + EPA (mg)	-	-	-	140	350	140%
**Sodium (g)**	0.05	0.125	10%	0.04	0.1	8.3%
**Vitamin D3 (μg)**	0.05	0.125	0.70%	7	17.5	100%
**Vitamin E (mg)**	0.03	0.075	0.60%	5	12.5	100%
**Vitamin C (mg)**	2.1	5.25	5.50%	19	47.5	50%
**Vitamin B6 (mg)**	0.036	0.09	5.60%	0.64	1.6	100%
**Vitamin B12 (μg)**	0.35	0.875	35%	1	2.5	100%
**Vitamin B9 (μg)**	7	17.5	4.40%	80	200	50%
**Selenium (μg)**	0.5	1.25	2.30%	22	55	100%
**Zinc (mg)**	0.35	0.875	8.75%	4	10	100%

LARN = Livelli di Assunzione di Riferimento di Nutrienti ed energia per la popolazione italiana, DHA = docosahexaenoic acid, EPA = eicosapentaenoic acid.

**Table 3:** Baseline characteristics of the study population.

Characteristics	Baseline FP vs. PF		q Value
Age, years	71 ± 4	70 ± 5	0.85
Gender			
Men, *n* (%)	13 (54)	13 (54)	-
Women, *n* (%)	11 (46)	11 (46)	
Height, cm	165 ± 0.1	165 ± 0.1	0.99
Weight, kg	78± 16	72 ±11	0.65
BMI, kg/m^2^	28 ± 5	26 ± 4	0.65
Waist circumference, cm	99 ± 16	93 ± 12	0.67
Hip circumference, cm	106 ± 9	101 ± 6	056
Waist/hip ratio, cm	0.9 ± 0.1	0.9 ± 0.1	0.95
Total cholesterol (mg/100 mL)	205 ± 36	215 ± 43	0.85
HDL cholesterol (mg/100 mL)	56 ± 13	56 ± 11	0.95
LDL cholesterol (mg/100 mL)	125 ± 30	128 ± 26	0.99
Triglycerides (mg/100 mL)	111 ± 34	133 ± 85	0.77
Glycaemia (mg/dL)	100 ± 15	103 ± 32	0.92
HOMA-IR	2 ± 2	3 ± 2	0.92
Creatinine (mg/dL)	1 ± 0.2	1 ± 0.2	0.99
Fibrinogen (mg/dL)	294 ± 46	299 ± 42	0.99
ALT (U/L)	18 ± 7	18 ± 8	0.99
ASP (U/L)	19 ± 4	20 ± 5	0.99

Data are reported as mean ± SD. The comparison between the two arms (FP vs. PF) was performed by using one-way ANOVA test with Benjamini–Hochberg correction, considering q (corrected p-value) < 0.05 statistically significant. No significant difference emerged for all the parameters considered. FP = arm: participants on fortified milk and then placebo, PF = arm: participants on placebo and then fortified milk, BMI = body mass index, HOMA-IR = homeostasis model assessment of insulin resistance, ALT = alanine aminotransferase, ASP = aspartate aminotransferase.

**Table 4:** Measurement of micronutrient levels in both arms at baseline and after washout.

Micronutrients	Baseline FP vs. PF		q Value	After Washout FP vs. PF		q Value
Vitamin B9 (ng/mL)	7 ± 4	7 ± 3	0.96	7.6 ± 3	6 ± 2	0.44
Vitamin B6 (µg/L)	14 ± 9	15 ± 6	0.70	18 ± 5	20 ± 25	0.96
Vitamin B12 (pg/mL)	376 ± 174	385 ± 125	0.96	339 ± 109	352 ± 154	0.96
Vitamin 25 (OH)D (ng/mL)	16 ± 11	15 ± 6	0.96	23 ± 9	20 ± 5	0.70
Vitamin E (µg/mL)	11 ± 3	13 ± 3	0.68	14 ± 3	12 ± 2	0.40
Zinc (µg/L)	809 ± 89	791 ± 172	0.96	799 ± 87	801 ± 97	0.96
Selenium (µg/L)	96 ± 11	97 ± 9	0.96	108 ± 14	108 ± 11	0.96
DHA (% in e.m.)	5 ± 1	5 ± 1	0.96	5.4 ± 1	4.4 ± 1	0.289
EPA (% in e.m.)	0.9 ± 0.4	0.9 ± 0.1	0.96	0.7 ± 0.3	0.8 ± 0.4	0.96

Circulating levels of the micronutrients are shown, except for the ω-3 DHA and EPA, which were detected in the erythrocyte membranes. Data are reported as mean ± SD. The comparison between the two arms (FP vs. PF) was performed at baseline and after the washout by using one-way ANOVA test with Benjamini–Hochberg correction, considering q (corrected p-value) < 0.05 statistically significant. FP = arm: participants on fortified milk and then placebo, PF = arm: participants on placebo and then fortified milk, DHA = docosahexaenoic acid, EPA = eicosapentaenoic acid, e.m. = erythrocyte membranes.

**Table 5:** Measurement of circulating micronutrient levels at baseline and after treatment in all participants.

Micronutrients	Reference Range	Baseline	After Fortified Milk	95% CI	q Value
Vitamin B9 (ng/mL)	2.5–20	7 ± 3	13 ± 5	5 (4–6)	<0.0001 *
Vitamin B6 (µg/L)	3.6–18	16 ± 18	22 ± 10	4 (1–7)	0.015 *
Vitamin B12 (pg/mL)	200–910	378 ± 158	395 ± 153	52 (33–71)	0.0001 *
Vitamin 25(OH)D (ng/mL)	30–100	18 ± 10	23 ± 6	7 (5–9)	0.0002 *
Vitamin E (µg/mL)	3–12	12 ± 3	13 ± 3	1 (0–2)	0.110
Zinc (µg/L)	600–1200	805 ± 89	798 ± 102	−10 (−52–32)	0.786
Selenium (µg/L)	0–150	102 ± 14	108 ± 15	4 (0.3–7)	0.106

Data are reported as mean ± SD and 95% Confidence Interval (CI). The comparison between the two groups (placebo vs. fortified milk) was performed by using linear mixed effect model with Benjamini–Hochberg correction and considering q (corrected p-value) < 0.05 statistically significant (*).

**Table 6:** Measurement of fatty acid profile in erythrocyte membranes at baseline and after treatment.

Fatty Acids	Reference Range	Baseline	After Fortified Milk	95% CI	q Value
Linoleic acid (%)	9–16	11 ± 1	11 ± 1	0 (−1–0)	0.058
DGLA (%)	1.9–2.4	2 ± 0.6	2 ± 0.3	−0.1 (−0.3–0)	0.190
Arachidonic acid (%)	13–17	16.3 ± 1	15.9 ± 2	−1 (−1–0)	0.001 *
TFA (%)	0–0.4	0.2 ± 0.1	0.3 ± 0.1	0.02 (−0.03–0.07)	0.559
SFA (%)	30–45	45 ± 2	45 ± 2	−0.1 (−0.8–0.6)	0.870
MUFA (%)	13–23	19 ± 1	19 ± 2	−0.1 (−0.5–0.3)	0.766
PUFA (%)	28–39	35 ± 2	36 ± 2	0.1 (−0.7–0.9)	
DHA (% in e. m.)	5–7	5 ± 1	6 ± 1	1 (1–2)	<0.0001 *
EPA (% in e. m.)	0.5–0.9	0.9 ± 0.4	1.1 ± 0.3	0.2 (0–0.3)	0.040 *

Data are reported as mean ± SD and 95% Confidence Interval (CI). The comparison between the two groups (placebo vs. fortified milk) was performed by using linear effect mixed model with Benjamini–Hochberg correction and considering q (corrected p-value) < 0.05 statistically significant (*). DGLA = dihomo-γ-linolenic acid, TFA = trans-unsaturated fatty acids, SFA = saturated fatty acids, MUFA = monounsaturated fatty acids, PUFA = polyunsaturated fatty acids, DHA = docosahexaenoic acid, EPA = eicosapentaenoic acid, e. m. = erythrocyte membranes.

## Supplementary Materials

The following are available online at https://www.mdpi.com/2072-6643/12/11/3580/s1, Figure S1: standardized Mini-Mental State Examination (SMMSE), Table S1: evaluation of the hematological-biochemical parameters at baseline and after the treatment, Table S2: evaluation of inflammatory parameters at baseline and after the treatment, Table S3: main measured parameters in each time point by treatment.
