# Supplementary material for: Twelve-Week Daily Consumption of ad hoc Fortified Milk with ω-3, D, and Group B Vitamins Has a Positive Impact on Inflammaging Parameters: A Randomized Cross-Over Trial
Source: Nutrients. 2020 Nov 22;12(11):3580. doi: 10.3390/nu12113580 (PMC7700442; doi:10.3390/nu12113580)
Supplement: Supplementary file 1 [file nutrients-12-03580-s001.pdf]

Supplementary material

Table S1 Evaluation of the hematological-biochemical parameters at baseline and after the treatment

**Parameters**	**Normal range**	**Baseline**	**After fortified milk**	**95% CI**	**q** Value
Total cholesterol (mg/100mL)	130-200	209 ± 36	216 ± 43	7 (0 - 13)	0.133
HDL cholesterol (mg/100mL)	≥ 39	55 ± 12	55 ± 12	0 (-1 - 1)	0.981
LDL cholesterol (mg/100mL)	≤ 130	127 ± 28	132 ± 30	5 (-1 - 11)	0.195
Triglycerides (mg/100mL)	35-180	114 ± 45	108 ± 42	-11 (-23 - 1)	0.169
Glycaemia (mg/dL)	67-112	97 ± 15	102 ± 20	3 (-1 - 8)	0.307
HOMA-IR	0.22-2.5	2 ± 2	3 ± 2	0.2 (-0.2 - 0.6)	0.559
Creatinine (mg/dL)	0.6-1.2	1 ± 0.2	1 ± 0.2	0 (-0.03 - 0.02)	0.931
Fibrinogen (mg/dL)	150-400	313 ± 48	313 ± 47	-8 (-21 - 4)	0.356
ALT (U/L)	8-40	18 ± 7	20 ± 6	3 (1 - 5)	0.019*
ASP (U/L)	8-40	19 ± 5	21 ± 5	2 (1 - 3)	0.0006*

Data are reported as mean ± SD and 95% Confidence Interval (CI). The comparison between the two groups (placebo *versus* fortified milk) was performed by using linear mixed effect model with Benjamini-Hochberg correction, considering q < 0.05 (corrected p-value) statistically significant (*). HOMA-IR = homeostasis model assessment of insulin resistance, ALT = alanine aminotransferase, ASP = aspartate aminotransferase.

Table S2 Evaluation of inflammatory parameters at baseline and after the treatment

**Parameters**	**Normal range**	**Baseline**	**After fortified milk**	**95% CI**	**q** Value
Interleukin IL-6 (pg/mL)	-	2 (± 3)	1 (± 2)	0 (-0.5 - 0.5)	0.980
Interleukin IL-10 (pg/mL)	-	1.4 (± 4)	1.3 (± 4)	0 (-0.1 - 0.3)	0.431
CRP (mg/L)	0-6	3.2 (± 3)	3.4 (± 3)	0 (-1 - 0)	0.508

Data are reported as mean ± SD and 95% Confidence Interval (CI). The comparison between the two groups (placebo *versus* fortified milk) was performed by using linear mixed effect model with Benjamini-Hochberg correction, considering q < 0.05 (corrected p-value) statistically significant. CRP = C-reactive protein.

Table S3 Main measured parameters in each time point by treatment

Parameters	Baseline	After fortified milk	After washout	After placebo
Vitamin B9 (ng/mL)	7 ± 3	13 ± 5	7 ± 3	7 ± 3
Vitamin B6 (µg/L)	16 ± 18	22 ± 10	19 ± 18	21 ± 20
Vitamin B12 (pg/mL)	378 ± 158	395 ± 153	346 ± 133	379 ± 177
Vitamin 25(OH)D (ng/mL)	18 ± 10	23 ± 6	22 ± 8	16 ± 6
Vitamin E (µg/mL)	12 ± 3	13 ± 3	13 ± 3	12 ± 3
Zinc (µg/L)	805 ± 89	798 ± 102	800 ± 93	808 ± 81
Selenium (µg/L)	102 ± 14	108 ± 15	108 ±13	104 ± 14
Linoleic acid (%)	11 ± 1	11 ± 1	11 ± 1	11 ± 1
DGLA (%)	2 ± 0.6	2 ± 0.3	2 ± 0.4	2 ± 0.3
Arachidonic acid (%)	16.3 ± 1	15.9 ± 2	16.2 ± 1	16.7 ± 1
TFA (%)	0.2 ± 0.1	0.3 ± 0.1	0.2 ± 0.1	0.2 ± 0.1
SFA (%)	45 ± 2	45 ± 2	45 ± 2	45 ± 2
MUFA (%)	19 ± 1	19 ± 2	20 ± 2	19 ± 1
PUFA (%)	35 ± 2	36 ± 2	35 ± 2	36 ± 2
DHA (% in e. m.)	5 ± 1	6 ± 1	4.9 ± 1	4.9 ± 1
EPA (% in e. m.)	0.9 ± 0.4	1.1 ± 0.3	0.7 ± 0.3	0.9 ± 0.3
ω-6/ω-3 PUFA (%)	5 ± 1	4 ± 1	5.7 ± 2	5 ± 1.4
ω-3 index (%)	5.6 ± 1	7.3 ± 1	5.7 ± 2	5.8 ± 1
Homocysteine (µmol/L)	15.2 ± 6	11.6 ± 4	14.7 ± 6	14.7 ± 6
Total cholesterol (mg/100mL)	209 ± 36	216 ± 43	210 ± 31	209 ± 36
HDL cholesterol (mg/100mL)	55 ± 12	55 ± 12	53 ± 11	55 ± 12
LDL cholesterol (mg/100mL)	127 ± 28	132 ± 30	129 ± 24	127 ± 29
Triglycerides (mg/100mL)	114 ± 45	108 ± 42	116 ± 47	118 ± 53
Glycaemia (mg/dL)	97 ± 15	102 ± 20	95 ± 17	98 ± 16
HOMA-IR	2 ± 2	3 ± 2	2.6 ± 1	2.5 ± 2
Creatinine (mg/dL)	1 ± 0.2	1 ± 0.2	1 ± 0.2	1 ± 0.2
Fibrinogen (mg/dL)	313 ± 48	313 ± 47	330 ± 48	323 ± 63
ALT (U/L)	18 ± 7	20 ± 6	18 ± 7	18 ± 9
ASP (U/L)	19 ± 5	21 ± 5	20 ± 5	19 ± 4

Data are reported as mean ± SD. FP = arm: participants on fortified milk and then placebo, PF = arm: participants on placebo and then fortified milk, DGLA = Dihomo-γ-linolenic acid, TFA = Trans-unsaturated fatty acids, SFA = Saturated Fatty Acids, MUFA = Mono Unsaturated Fatty Acids, PUFA = Poly Unsaturated Fatty Acids, DHA = Docosahexaenoic acid, EPA = Eicosapentaenoic acid, e. m. = erythrocyte membranes, HOMA-IR = homeostasis model assessment of insulin resistance, ALT = alanine aminotransferase, ASP = aspartate aminotransferase.

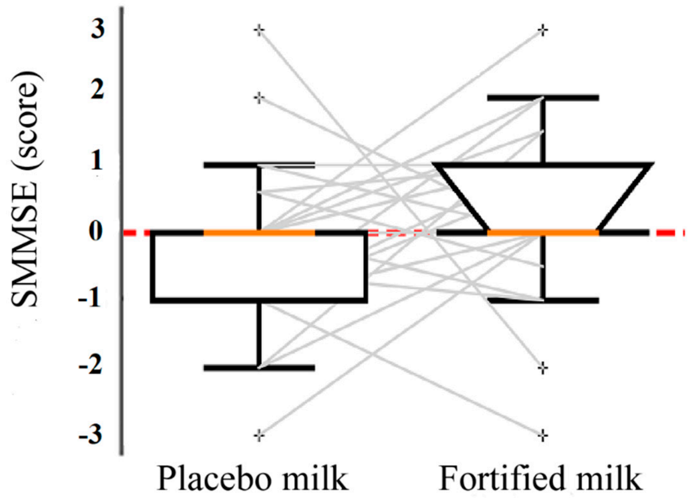

Figure S1. Standardized Mini-Mental State Examination (SMMSE). Boxplot of the variation of SMMSE scores in participants on placebo *versus* those on fortified milk are displayed. A not significant trend of the SMMSE score to increase after fortified milk consumption emerged (q = 0.749). Statistical analysis was performed by linear mixed model effect and a q-value (corrected p-value for the Benjamini-Hochberg multiple comparisons) < 0.05 was considered statistically significant.
